# A Developmental Approach to Machine Learning?

**DOI:** 10.3389/fpsyg.2017.02124

**Published:** 2017-12-05

**Authors:** Linda B. Smith, Lauren K. Slone

**Affiliations:** Department of Psychological and Brain Sciences, Indiana University Bloomington, Bloomington, IN, United States

**Keywords:** development, egocentric vision, object recognition, active vision, natural environment

## Abstract

Visual learning depends on both the algorithms and the training material. This essay considers the natural statistics of infant- and toddler-egocentric vision. These natural training sets for human visual object recognition are very different from the training data fed into machine vision systems. Rather than equal experiences with all kinds of things, toddlers experience extremely skewed distributions with many repeated occurrences of a very few things. And though highly variable when considered as a whole, individual views of things are experienced in a specific order – with slow, smooth visual changes moment-to-moment, and developmentally ordered transitions in scene content. We propose that the skewed, ordered, biased visual experiences of infants and toddlers are the training data that allow human learners to develop a way to recognize everything, both the pervasively present entities and the rarely encountered ones. The joint consideration of real-world statistics for learning by researchers of human and machine learning seems likely to bring advances in both disciplines.

## Introduction

Learning – *adaptive intelligent change* in response to experience – is a core property of human cognition and a long-sought goal of artificial intelligence. There is growing excitement (Cadieu et al., 2014; Kriegeskorte, 2015; Marblestone et al., 2016) that we are at the tipping point for powerful new insights into both human and artificial intelligence and that these insights will emerge more rapidly by explicitly connecting advances in human cognition, human neuroscience, and machine learning. ‘Thought-papers’ are making explicit calls to researchers in machine learning to use human and neural inspiration to build machines that learn like people (e.g., Kriegeskorte, 2015; Marblestone et al., 2016), and for researchers in human cognition and neuroscience to leverage machine learning algorithms as hypotheses about cognitive, visual and neural mechanisms (Yamins and DiCarlo, 2016). One impetus for this renewed interest is the remarkable successes of deep-learning networks to solve very hard – and sometimes previously unsolvable – learning problems (e.g., Silver et al., 2016). Of the lineage of neuron-inspired perceptrons and connectionist networks, deep-learning networks take raw sensory information as input and use multiple hierarchically organized layers with the output of each layer serving as the input to the next, resulting in a cascade of feature extraction and transformation. One domain in which these networks have been particularly successful is machine vision. The layered structure and spatial pooling of these convolutional deep learning networks (CNNs) not only yield state-of-the-art image recognition but do so via a hierarchical organization of feature extraction that approximates the functions of the cortical layers in the human visual system (Cadieu et al., 2014).

**FIGURE 1:**
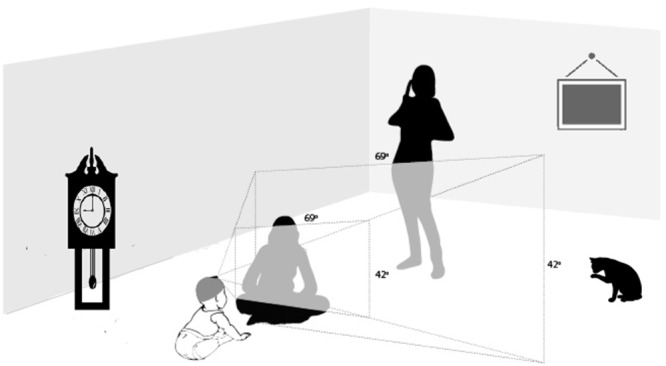
The selective nature of egocentric views. The field of view indicated with shading corresponds to the field of view of the infant’s head camera.

On the human cognition side, recent advances in head-mounted cameras and head-mounted eye-tracking technology have yielded exciting discoveries concerning natural learning environments. The structure and regularities in humans’ everyday visual environments – particularly those of infants and children – are not at all like the training sets used in state-of-the-art machine vision. The training images for machine learning are photographs taken and framed by adults. Thus, they are biased to “what looks good” for the mature system, reflecting the outcomes of perceptual development and not necessarily the scenes that drove that development (e.g., Fathi et al., 2011; Foulsham et al., 2011; Smith et al., 2015). Real world perceptual experience is not framed by a camera but is tied to the body as it acts in the world. As a consequence, the learner’s own view of the visual environment is highly selective, dependent on momentary location, orientation in space, posture, and head and eye movements (see Smith et al., 2015, for a review). The selectivity of the ego-centric view is illustrated in **Figure 1**: not everything in the immediate environment is in the infant’s view; unless the infant turns their head and looks, the cat, the window, the clock, the standing person’s face are not in view. The perceiver’s posture, location, movement, interests, and social interactions systematically bias the point-of-view visual information.

And all of these – posture, location, movement, interests – change dramatically with development biasing different classes of visual experience as the individual grows. Particularly, in the first 2 years of life, each new sensory-motor achievement – rolling over, reaching, crawling, walking (and more) – opens gates to new classes of visual experience. Thus, rather than batch processing, the human visual system develops through a systematically ordered curriculum of visual experience designed through the infants’ own sensory-motor development. Egocentric vision systems provide researchers with direct access to the properties of these developmentally constrained visual environments. Here, we consider the potential relevance for machine learning of these new findings about the data sets for real world visual learning.

One might ask, given all the successes of contemporary computer vision, why should machine learners care about how children do it? Schank, a seminal figure in the early days of artificial intelligence wrote: “We hope to be able to build a program that can learn, as a child does... instead of being spoon-fed the tremendous information necessary” (Schank, 1972). This would still seem a suitable goal for autonomous artificial intelligence. More recently, at a large machine learning conference, Malik (2016, personal communication, see also Agrawal et al., 2016) told young machine learners who wanted to be ready for the next big advances in machine learning to “go study developmental psychology seriously and then bring that knowledge in to build new and better algorithms.” With this in mind, we begin with an example of why machine learners should care about the regularities in children’s learning environments: a well-documented example of prowess in visual learning by human 2-year-olds that is as yet unmatched in contemporary computer vision (but see Ritter et al., 2017).

## What 2-Year-Olds Can Do

People can recognize a large number of instances of a very large number of categories and do so under varied conditions (Kourtzi and DiCarlo, 2006; Gauthier and Tarr, 2016). Recognizing all these instances and categories requires visual training; people have to see dogs, cars and toasters to visually recognize instances of those categories (e.g., Gauthier et al., 2000; Malt and Majid, 2013; Kovack-Lesh et al., 2014). This is true for people as well as computer vision algorithms. But the developmental trajectories for children and algorithms are currently quite different. For children, early learning is slow and error filled (e.g., MacNamara, 1982; Mervis et al., 1992). Indeed, 112-year-old children may well-perform worse in visual object recognition tasks than the best performing computer vision algorithm, as 112-year-old children’s category judgments are characterized by many over- and under-generalizations as well as sometimes complete failure to recognize known objects in visually crowded scenes (Farzin et al., 2010). However, this changes after the second birthday. At that point children can infer the extension of a whole category from one example. Given *just one instance* of a novel category, and its name, 2-year-old children immediately generalize that name in an adult-like manner. For example, if a 2-year-old child encounters their very first tractor – say, a green John Deere working in a field – while hearing its name, the child from that point forward will recognize all variety of tractors as tractors – red Massey-Fergusons, antique tractors, ride-on mowers – but not backhoes or trucks. This phenomenon, known as the “shape bias” in the developmental literature is an example of “one-shot” learning that has been observed in children’s natural category learning and has been replicated and extensively studied in the laboratory (e.g., Rosch et al., 1976; Landau et al., 1988; Samuelson and Smith, 2005).

We know a great deal about the “shape bias” and its development. We know that the emergence of the shape bias co-occurs with rapid growth in children’s object name vocabularies. We know that the bias is about the perceived shapes of things and emerges when children can recognize known objects from the relational structure of the major parts (Gershkoff-Stowe and Smith, 2004). We know the shape bias is itself learned as a consequence of the slow learning of an initial set of object names (50 to 150 learned categories by some estimates, Gershkoff-Stowe and Smith, 2004). We know that early intensive training of shape-based object categories in the context of object play causes an earlier than typical emergence of the shape bias in 112-year-olds, and an early increase in the rate of growth of these children’s vocabularies (Samuelson, 2002; Smith et al., 2002; Yoshida and Smith, 2005; Perry et al., 2010). We also know that the shape bias co-develops not just with children’s learning of object names but also with object manipulation (Smith, 2005; James et al., 2014a), and with children’s emerging ability to recognize objects from abstract representations of 3-dimensional shape (Smith, 2003, 2013; Yee et al., 2012). We know that children who have difficulty in learning language – late-talkers, children with specific language impairment, children with autism – do not develop a robust shape bias (Jones, 2003; Jones and Smith, 2005; Tek et al., 2008; Collisson et al., 2015; Potrzeba et al., 2015). In brief, typically developing children, over the course of slowly learning the names for an initial set of object categories, learn how to visually represent object shape in a way that enables them to approximate the boundaries for novel object categories given just a single instance of that category. State-of-the-art machine vision operates differently. There is no learning to learn that shifts the very nature of learning itself. Instead, each to-be-learned category requires extensive training with many examples.

Wherein lies the difference? All learning depends on both the learning machinery and the training data. Toddlers are highly successful learners of visual categories; thus, their internal algorithms must be able to exploit the regularities in their everyday experiences, whatever those regularities are. Therefore, understanding infants’ everyday visual environments – and how they change with development – not only helps to reveal the relevant training data, but also provides information about the internal machinery that does the learning.

## Developmentally Changing Visual Environments

**FIGURE 2:**
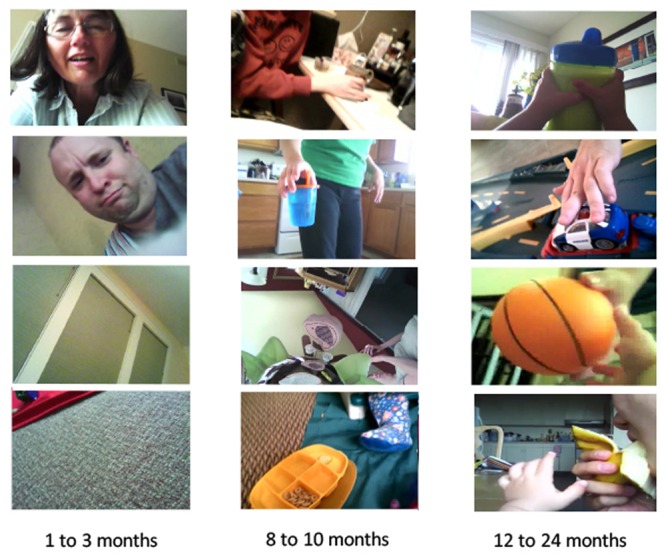
Sample head-camera captured images for three different ages of infants.

The data from infant head camera studies are quite clear: the training sets for human visual learning change substantially with development. Example head-camera captured images are shown in **Figure 2**. One example concerns infants’ egocentric views of the people around them. Analyses of a large corpus of head camera images collected by infants as they went about their daily experiences (Jayaraman et al., 2015, 2017; Fausey et al., 2016) show that people are persistently in infant head-camera images and are so at the same rate for newborn infants and for 2-year-olds. This is not surprising as infants and toddlers cannot be left alone. However, the specific body parts in the head camera images of younger and older infants are not the same. For infants under 3 months of age, human faces are pervasively present, with faces constituting more than 15 min out of every hour of visual experience. Moreover, these faces are consistently close to the young infant (within 2 feet of the head camera) and show both eyes. By the time infants near their first birthday, however, faces are rare in the recorded head camera images, present for about only 6 min out of every waking hour. Instead, for 1- and 2-year-olds, other people’s hands are in view (Fausey et al., 2016). These hands are predominantly (in over 85% of all images with hands) in contact with and manipulating an object. This shift in the contents of the visual scenes in front of infants is driven by changes in their sensory-motor abilities, by the corresponding behavior of parents, and by changes in infant interests. The products of all these interconnected forces are the data for visual learning, and the data change – from many full view and close faces to many hands acting on objects. We strongly suspect this order – early faces, later objects – matters to how and why human visual object recognition develops the way it does.

The importance of early visual experiences dense with faces is indicated by sleeper effects in configural face processing. Maurer et al. (2007) defined a sleeper effect as a permanent deficit that emerges late in development but that results from an *early* deficit in experience. One example concerns the case of infants deprived of early visual input by congenital cataracts that were removed by the time the infants were just 2 to 6 months of age. By multiple benchmarks of visual development (including acuity, contrast sensitivity) these infants, after removal of the cataracts, caught up to their peers and showed a typical trajectory of visual development. But as adults these individuals show a permanent deficit in one of the defining mature properties of human visual face processing: configural face processing. Configural processing refers to the discrimination and recognition of individual faces based on a gestalt-like representation that suppresses information about individual features. This is an aspect of human visual processing that does not begin to emerge until relatively late, around 5 to 7 years of age (Mondloch et al., 2002). Maurer et al. (2007) hypothesized that early experiences preserve and/or establish the neural substrate for face-processing abilities that develop much later (see also Byrge et al., 2014). We conjecture that the dense experience of close, full-view faces by very young infants is the missing component of the very early experiences of infants with congenital cataracts. Because these experiences are tied to the infant’s own changing biases and sensorimotor skills, they will not be replaced by their social partners when the infant’s cataracts are later removed because by that time the infant’s own behaviors and autonomy will create very different social interactions. By hypothesis, then, dense *early* experiences with faces may be necessary to set up or maintain the cortical circuitry that supports the *later* emergence of specialized face processing.

It could be the case that early face experiences are only important for face processing, a domain-specific experience for a domain-specific outcome. However, there is a case to be made for an alternative view. The human visual cortex builds the world we see through a hierarchical system of successive feature extractions and transformations (e.g., Hochstein and Ahissar, 2002). All input goes through and tunes the same lower layers and all higher layers of representations – faces, objects, letters – compute over the activity of lower layers. In this way, learning about faces and *learning about non-face object categories* both depend on the precision, tuning, and activation patterns of the same lower layers. Simple visual discriminations at lower layers can have far-reaching generality across higher level visual processes (e.g., Ahissar and Hochstein, 1997). The head-camera images from human infants indicate that the initial tuning and development of the lower layers is done through visual scenes that include many close faces with two eyes in view. Because of this, children’s later learning and extraction of features of non-face objects will be shaped at least in part by an early tuning of lower layers, tuning heavily biased by the low-level visual features of up-close faces.

Although Maurer et al. (2007) used the term sleeper effects to refer to *deficits* in experience, the role of early visual experience on later emerging achievements may be conceptualized both negatively and positively. Regularities in an individual’s early experiences will train and tune layers in this hierarchical system, and in so doing may set up potentially hidden competencies, that are critical to and play out in later learning. Research on human development provides many unexplained examples of the far reach of past learning into future learning. For example, the precision of visual discrimination of dot arrays predicts later mathematics achievement (Halberda et al., 2008) and the shape bias in toddlers predicts the ability to learn letters (Augustine et al., 2015; see also Zorzi et al., 2013). Similar to the human visual system, deep learning networks are “deep” in that they contain a hierarchical cascade of layers. This structure means that, similar to human vision, the early layer representations formed in one task will be reused and in principle can influence – both negatively and positively – the solutions that are found in learning in other tasks. The computational value of ordered training sets for such hierarchically layered learning systems is not yet well-understood. Could the whole curriculum of developmentally constrained training sets – from faces to hands on objects – be part of the complete explanation of how 2-year-olds seem to know the boundaries of non-face object categories from just one or a few instances?

## Learning A Lot About A Few Things

**FIGURE 3:**
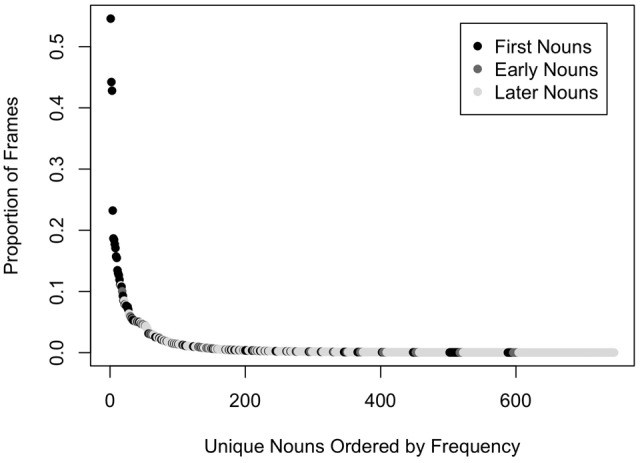
The distribution of common object categories in head camera images of 8- to 10-month-old infants (Clerkin et al., 2017). Object categories are colored based on norms for their age of acquisition (Fenson et al., 1994): first nouns (object names that are in the receptive vocabulary of at least 50% of 16-month-old infants), early nouns (object names that are not first nouns and in the productive vocabularies of at least 50% of 30-month-old children), and later nouns (all other object names).

Analyses of head camera images from infants in the first 2 years of life also tell us that the distribution of entities in these images is neither a random sample of entities in the world nor are the entities present in these egocentric images uniformly distributed. Instead, experience is extremely right-skewed. The objects in infants’ head camera images are highly selective – *a very few kinds* are pervasive and most things are rare. Here is a key question then: how does extensive (and potentially slow) learning about a few things yield a learning system that can rapidly learn about all those individually rare things? A power-law-like distribution characterizes both infants’ experiences of unique individual’s faces (Jayaraman et al., 2015) and their experiences of objects (Clerkin et al., 2017). Throughout the whole first year of their lives, infants see the faces of a very few people repeatedly, with the three most frequent individuals accounting for about 80% of all faces in the head camera images. Likewise, the objects in infants’ visual environments are also extremely right skewed, with some object categories much more frequent that others (Clerkin et al., 2017). **Figure 3** shows the distribution of common object categories in one analysis of head camera images for 8- to 10-month-old infants across 147 unique meal-time events (Clerkin et al., 2017). A very few object categories are pervasively present while most are very rare. Intriguingly, the most frequently encountered object categories have names that are also acquired very early, but later than 8 to 10 months, just after the first birthday, suggesting that dense early visual experiences prepare the system for later learning of these specific objects’ labels.

One possible advantage of extremely right-skewed distributions is that the pervasiveness of a relatively small set of individual objects and object categories enables the infant to define an initial target set for learning (Clerkin et al., 2017; see also Salakhutdinov et al., 2011) and then to master the *visual invariances* relevant to recognizing these few objects across many different viewing conditions. This may be a key step – complete learning about a few things – that then leads to generalized competencies that enable rapid learning from limited experience, such as seen in the shape bias in 2-year-old children (Smith, 2013). This complete learning about a very few things may depend on not just many experiences but extended experiences in time. When a single object is viewed for an extended time, the retinal information with respect to that object will necessarily and continuously change, revealing relevant transformations and the invariances for recognition that may be extendable to recognizing novel things (Földiák, 1991; Wiskott and Sejnowski, 2002; Li and DiCarlo, 2008).

Research with controlled-reared chicks (Wood, 2013; Wood and Wood, 2016) provides a demonstration proof of this idea: slow-changing transformations of objects provide sufficient input for generalized learning by chicks about object shape. In these studies, newborn chicks were raised in tightly controlled visual environments and shown moving and rotating individual objects. Across a series of controlled-rearing experiences, the properties of movement and rotation were experimentally manipulated. The results show that experiences of a single object seen over time are sufficient for chicks to build robust object recognition skills that generalize to unseen views of that object and unseen objects (Wood, 2013, 2015). The controlled rearing experiments (Wood, 2016; Wood et al., 2016) also indicate two major constraints on chick learning: slowness and smoothness. Changes in object views needed to occur slowly and smoothly, *adhering to the spatiotemporal properties of a physical object in the world.* Chickens have very different brains and visual systems than humans and thus the relevance of the chick data is not that of an animal model of the human visual system. Rather, the relevance of these findings is that they clearly show useable information in temporally sustained experience with a single visual object and by implication indicate as-yet unspecified algorithms that could rapidly learn to recognize object categories from extended visual experiences with very few, perhaps just one, object.

## Self-Generated Visual Experiences

Toddlers’ knowledge of object names is measured either by their choice of a referent when asked for an object by name or by their spontaneous production of an object name upon visually encountering an object. Toddlers’ object name vocabulary is thus a good proxy for measuring their ability to visually recognize objects. Object name learning begins very slowly prior to the first birthday, with children’s knowledge of individual object names growing incrementally and initially characterized by errors (e.g., MacNamara, 1982; Mervis et al., 1992, see also Bloom, 2000). Around 18 to 24 months (with the timing different for different children), the character and rate of learning changes. Around 2 years of age, object name learning becomes seemingly effortless as typically developing children need very little experience, often just a single experience with a named object, to generalize the name appropriately to new instances (Landau et al., 1988; Smith, 2003). The shift from slow incremental learning to rapid nearly “one-shot” learning reflects changes in the internal machinery brought on by learning itself (Smith et al., 2002). However, growing evidence indicates that there is also a dramatic change in the visual data for learning.

For 8- to 10-month-old infants, the scenes captured by head cameras are often cluttered (Clerkin et al., 2017). After 12 months scenes are still often cluttered, but these are punctuated by sustained series of scenes in which just one object visually dominates (e.g., Yu and Smith, 2012). The change in scene composition is the direct consequence of infants’ developing manual skills. Well-before their first birthday, infants reach for and hold objects but they lack the trunk stability required for long engaged play sessions (Rochat, 1992; Soska et al., 2010) and they lack the manual skills to rotate, stack or insert objects (Pereira et al., 2010; Street et al., 2011). Further, they are mostly interested in putting objects in their mouths which is not ideal for visual learning. As a consequence, they often look at the world from afar and from afar the world is cluttered with many things. After their first birthday, all this changes. Toddlers view objects up close while actively handling them. This manual activity supports improved visual object memory and discrimination (Ruff, 1984; Soska et al., 2010; Möhring and Frick, 2013; James et al., 2014a) and object-name learning (e.g., Yu and Smith, 2012; LeBarton and Iverson, 2013; James et al., 2014a). There are three properties of toddlers’ self-generated object views that likely underlie these advances.

First, toddlers’ handling of objects creates visual scenes that are less cluttered than those of younger infants (Yu and Smith, 2012; Clerkin et al., 2017) and also of adults (Smith et al., 2011; Yu and Smith, 2012). Toddlers have short arms and lean in to look closely at handled objects. In so doing, they create scenes in which one object fills the visual field. This solves many fundamental problems including segmentation, competition, and referential ambiguity. One study (Bambach et al., 2017) directly compared how well a commonly used CNN (Simonyan and Zisserman, 2014) could learn to recognize objects given training sets consisting of toddler versus adult head camera images (of the same real world events). The network was not presented cropped images of the to-be-trained object, but whole scenes, with no information about the relevant location of the target object in the scene. Learning was more robust and showed better generalization given toddler than adult scenes. This fits contemporary practices in computer vision, which commonly feed their algorithms cropped images or scenes with bounding boxes to specify the object for learning. Toddlers use their own hands and heads to do this.

**FIGURE 4:**
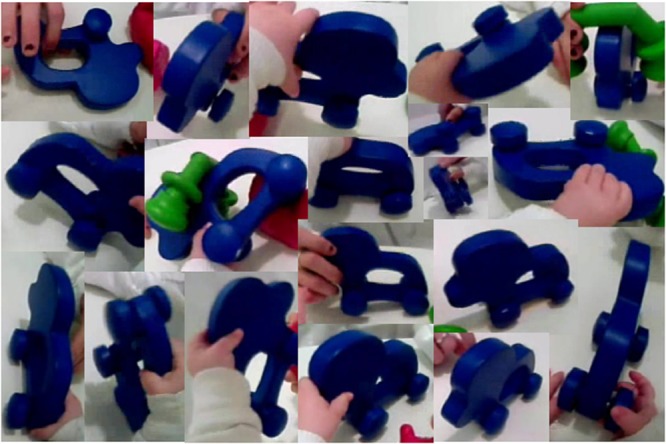
Sample images of a single object captured by a 15-month-old infant’s head-camera during play.

A second property of toddlers’ handling of objects is that they generate highly variable images of a single object. **Figure 4** shows an assortment of views of a single object generated by one 15-month-old toddler during play (Slone et al., under review). In this study, head-mounted eye-trackers were used to capture fixated object in the first-person scenes. A single algorithmic measure, mask orientation (MO), was used to capture the frame-by-frame *image* variability of objects on which infants fixated their gaze: MO is the orientation of the most elongated axis of the object pixels in the image. Critically, this is not a measure of the real-world orientation or shape of the object, nor does it relate in any direct way to the shape properties of the distal stimulus, but is instead a measure of the proximal image properties from which the visual system must determine the distal object. The main result is this: the amount of variability in MO generated by an infant during toy play at 15 months predicted infant object-name vocabulary 6 months later, when the infants were 21 months of age. In brief, greater variability led to better learning. In a related computational study (Bambach et al., 2017), a CNN was fed training sets consisting of images *of a joint play event* captured from either parent- or toddler-worn head cameras. The more variable object images from the toddler-worn camera led to more robust learning and generalization of that learning than did the less variable views of the same objects from the parent-worn cameras. These findings should change how we think of one-shot learning. Toddler’s visual experience with one object is not a single experience but a series of very different views of the same things. Could this series of different views of a single instance (for example, the John Deere working in the field) lead young learners to the generative principle that enables recognition of all members (for example, tractors in general)?

A third property of toddler self-generated object views is that they are biased (Pereira et al., 2010) toward views in which the most elongated axis of the object is perpendicular to the line of sight (easiest way to hold) and also (albeit more weakly) to views in which the most elongated axis is parallel to the line of sight (easiest way to insert a held object into another). Toddlers transition between these favored views by rotating the major axis of the object in depth. These biased views and the in-depth rotations highlight non-accidental shape features. The biases created by how hands hold objects may have a visual source as well in that they are stronger when toddlers hold and view objects contained in transparent spheres (James et al., 2014b) such that all views are equipotential with respect to the hands. Neither the right analyses nor the right experiments have been done to compare the properties of these self-generated views of objects to the smoothness and slow-change constraints proposed by Wood (2016) in his studies of chicks. But given the spatio-temporal constraints of the physical world and physical bodies, there is every reason to believe toddler self-generated views will comply.

Toddlers’ whole-body approach to seeing creates unique visual training sets that seem structured to teach a very specific lesson: view-independent recognition of three-dimensional shape. The single object is visually isolated in the image because it fills the image. The different views are connected to each other by their proximity in time and by hand contact, which provides a potent learning signal that two different views belong to the same object. The dynamic structure of the views highlights non-accidental shape properties. Here is a hard problem in visual object recognition that may be solved pretty much by the structure in the data itself.

## From Development to Machine Learning and Back

The visual environments of infants and toddlers change with development, segregating and ordering different learning tasks, such that later learning may build on prior learning in a different domain. Within each domain, the training sets concentrate on a limited sample of individual entities – the faces of 2 to 3 individuals, a small set of pervasive objects, many views of a single thing – but from these experiences builds general knowledge of how to recognize and learn about many different kinds of things. This is not a case of learning from limited data; the data are massive – about your mother’s face, about all the views of your sippy cup. The overall structure of these training sets are very different from those commonly used in computer vision. Could they be part of a next advance in more powerful machine learning?

Machine learning has made enormous strides without taking a developmental multistage approach to training. It is arguable that learning machines that do not require this tutoring and structured curricula are more powerful. Indeed, connectionist theories of linguistic development that used ordered training sets (Rumelhart and McClelland, 1986) and added difficulty as learning progressed were strongly criticized as cheating (Pinker and Prince, 1988). But, the criticized idea is the developmentally right one (Elman, 1993). There are current approaches to machine learning (curriculum learning, and iterative teaching, for example) that seek to optimize learning through ordered and structured training sets (e.g., Bengio et al., 2009; Krueger and Dayan, 2009). These efforts have not worried much about the structure in the natural learning environments of infants; it might be a useful convergence of human and machine learning to do so. The data for infant and toddler learning, however, are not just ordered over developmental time, but are also dynamically structured in real time by the learners’ own activity. The input at any moment depends on the current state of the learner, and will in real time change as the learner’s internal system changes as a function of learning. In this way, the information provided at any point in time may be optimal for the current state of learning, providing just the right information at the right time. One current relevant approach in machine learning trains attention in deep networks during the course of learning so that the data selected for learning changes with learning (Mnih et al., 2014; Gregor et al., 2015). Another approach uses curiosity to shift attention to new learning problems as learning progresses (Oudeyer, 2004; Houthooft et al., 2016; see also Kidd and Hayden, 2015). How can we foster the incorporation of developmental insights into machine learning? In considering the case of how a machine learner might progress from a slow and incremental learner to a “one-shot” learner with a shape bias of the kind shown by children, Ritter et al. (2017) “cognitive psychology” experiments on machine learners. Such experiments might manipulate both the structures of training sets (see Liu et al., 2017) as well as the algorithms to understand how early learning constrains later learning and how learning a lot about a very little may yield more generalized and powerful learning than learning a little about a lot of things.

There is, of course, no guarantee that by pursuing these ideas that machine learners will build powerful algorithms that can win current competitions. But, it seems certain that such an effort would yield new principles of learning. These principles –expressed in algorithmic form – would constitute a great advance in understanding human learning and intelligence.

## Author Contributions

All authors listed have made a substantial, direct and intellectual contribution to the work, and approved it for publication.

## Conflict of Interest Statement

The authors declare that the research was conducted in the absence of any commercial or financial relationships that could be construed as a potential conflict of interest.
